# Benzyl 2-ethyl­hexyl sulfoxide

**DOI:** 10.1107/S1600536809044328

**Published:** 2009-10-31

**Authors:** Xu Zhi-Guang, Liu Hai-Yang, Gu Guo-Bang, Xu Xuan, Zeng Yun-Xiu

**Affiliations:** a School of Chemistry and Environment, South China Normal University, Guangzhou 510006, People’s Republic of China; b Department of Chemistry, South China University of Technology, Guangzhou 510641, People’s Republic of China

## Abstract

The mol­ecule of the title compound, C_15_H_24_OS, shows *S* conformations for the S atom and the asymmetric C atom of the isooctyl group. The long axes of the mol­ecules are directed along the *c* axis. In the crystal structure, the mol­ecules are linked by weak inter­molecular bifurcated C—H⋯O hydrogen bonds.

## Related literature

For an X-ray and neutron diffraction study of benzyl *tert*-butyl sulfoxide, see: Iitaka *et al.* (1986 ▶). For an X-ray study of a flexible disulfoxide ligand, 1,6-bis­(benzyl­sulfin­yl)hexane, see: Li *et al.*, (2003 ▶); For the use of sulfoxides in the separation of palladium from other platinum-group metals by solvent extraction, see: Xu *et al.* (2006 ▶, 2007 ▶).
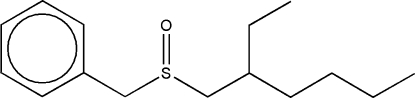


## Experimental

### 

#### Crystal data

C_15_H_24_OS
*M* *_r_* = 252.41
Monoclinic, 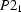

*a* = 8.832 (2) Å
*b* = 5.2321 (14) Å
*c* = 16.588 (4) Å
β = 102.005 (3)°
*V* = 749.8 (3) Å^3^
*Z* = 2
Mo *K*α radiation
μ = 0.20 mm^−1^
*T* = 273 K
0.26 × 0.22 × 0.15 mm

#### Data collection

Bruker SMART APEXII diffractometer
Absorption correction: multi-scan (*SADABS*; Bruker, 2005 ▶) *T*_min_ = 0.949, *T*_max_ = 0.970
4539 measured reflections
3119 independent reflections
2527 reflections with *I* > 2σ(*I*)
*R*_int_ = 0.021

#### Refinement

*R*[*F* ^2^ > 2σ(*F* ^2^)] = 0.039
*wR*(*F* ^2^) = 0.095
*S* = 1.06
3119 reflections
156 parameters
1 restraint
H-atom parameters constrained
Δρ_max_ = 0.15 e Å^−3^
Δρ_min_ = −0.15 e Å^−3^
Absolute structure: Flack (1983 ▶), 1074 Friedel pairs
Flack parameter: −0.03 (8)

### 

Data collection: *APEX2* (Bruker, 2005 ▶); cell refinement: *SAINT* (Bruker, 2005 ▶); data reduction: *SAINT*; program(s) used to solve structure: *SHELXS97* (Sheldrick, 2008 ▶); program(s) used to refine structure: *SHELXL97* (Sheldrick, 2008 ▶); molecular graphics: *ORTEP-3 for Windows* (Farrugia, 1997 ▶); software used to prepare material for publication: *SHELXL97* and *PLATON* (Spek, 2009 ▶).

## Supplementary Material

Crystal structure: contains datablocks I, global. DOI: 10.1107/S1600536809044328/si2215sup1.cif

Structure factors: contains datablocks I. DOI: 10.1107/S1600536809044328/si2215Isup2.hkl

Additional supplementary materials:  crystallographic information; 3D view; checkCIF report

**Fig. 2.**
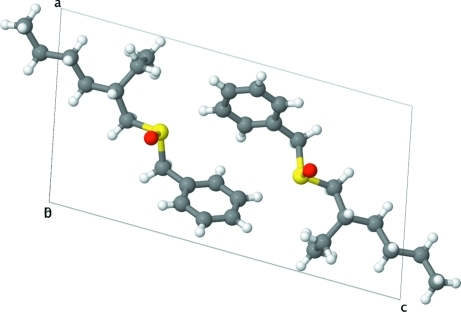
Molecular packing of the title compound as viewed along the b axis.

Enhanced figure: interactive version of Fig. 2

## Figures and Tables

**Table 1:** Hydrogen-bond geometry (Å, °)

*D*—H⋯*A*	*D*—H	H⋯*A*	*D*⋯*A*	*D*—H⋯*A*
C8—H8*A*⋯O1^i^	0.97	2.39	3.258 (3)	149
C1—H1*B*⋯O1^i^	0.97	2.49	3.333 (3)	145

Symmetry code: (i) 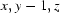.

**Fig. 1.**
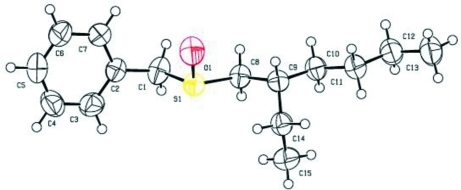
Molecule structure of (I) with displacement ellipsoids drawn at the 50% probability level.

## supplementary crystallographic information

### Comment

Sulfoxides have been widely used in the separation of palladium from other platinum-group metals(PGMs) by solvent extraction (Xu *et al.*, 2006). The experimental results indicated that the title compound exhibited excellent extraction property to PGMs (Xu *et al.*,2007). A similar disulfoxide ligand 1,6-bis(benzylsulfinyl)hexane and its Copper(II) and Cadmium(II) dimeric complexes were obtained (Li *et al.*,2003).

The stucture of the title compound, (I), Fig.1, exhibit the *S* conformation for the sulfur atom and asymmetric carbon atom of the isooctyl group. The long axes of the molecules are directed along the *c* axis. Additionally, the crystal structure exhibits weak intermolecular bifurcated C—H···O hydrogen bonds (for geometric details see Table 1).

### Experimental

The title compound was prepared refering to the literature method (Li *et al.*,2003; Iitaka *et al.*, 1986) with little modification. Sodium hydroxide (99%, 0.273 g, 0.0068 mol) and 1-isooctyl mercaptan (1.000 g, 0.0068 mol) were dissolved in anhydrous ethanol (50 ml) at 70°C, and then benzylchloride (0.86 g, 0.0068 mol) was added to the above solution with stirring over 1 h. The solution was extracted with CH_2_Cl_2_ after addition 400 ml of water. Benzyl isooctyl sulfide(1.412 g, 0.0060 mol) was obtained after evaporation of CH_2_Cl_2_. Yield: 87%. Hydrogen peroxide (30%, 0.0043 mol) was added dropwise to a solution of benzyl isooctyl sulfide (1.000 g, 0.0042 mol) in acetic acid (60 ml) on ice bath with a vigorously stir for 1 h. 500 ml of water was added. The solution was extracted with CH_2_Cl_2_, and the product of benzyl isooctyl sulfoxide(0.943 g, 0.0037 mol) was obtained after evaporation of CH_2_Cl_2_. Yield: 88%. It was characterized by recording its infrared and NMR spectra. White single crystals of the title compound were obtained by slow evaporation of its mixed solution including n-hexane and dichloromethane.

### Refinement

All H atoms were placed in calculated positions and subsequently constrained to ride on their parent atoms, with C–H distances of 0.93 Å (C-aromatic) and 0.97 Å (*C*-methyl). The *U*_iso_(H) values were set at 1.2 *U*_eq_(C aromatic) and 1.5 *U*_eq_(C methyl).

### Crystal data

**Table:**

C_15_H_24_OS	*F*(000) = 276
*M**_r_* = 252.41	*D*_x_ = 1.118 Mg m^−^^3^
Monoclinic, *P*2_1_	Mo *K*α radiation, λ = 0.71073 Å
Hall symbol: P 2yb	Cell parameters from 2250 reflections
*a* = 8.832 (2) Å	θ = 2.4–23.4°
*b* = 5.2321 (14) Å	µ = 0.20 mm^−^^1^
*c* = 16.588 (4) Å	*T* = 273 K
β = 102.005 (3)°	Block, white
*V* = 749.8 (3) Å^3^	0.26 × 0.22 × 0.15 mm
*Z* = 2	

### Data collection

**Table:**

Bruker SMART APEXII diffractometer	3119 independent reflections
Radiation source: fine-focus sealed tube	2527 reflections with *I* > 2σ(*I*)
graphite	*R*_int_ = 0.021
φ and ω scans	θ_max_ = 28.3°, θ_min_ = 2.4°
Absorption correction: multi-scan (*SADABS*; Bruker, 2005)	*h* = −7→11
*T*_min_ = 0.949, *T*_max_ = 0.970	*k* = −6→6
4539 measured reflections	*l* = −21→22

### Refinement

**Table:**

Refinement on *F*^2^	Secondary atom site location: difference Fourier map
Least-squares matrix: full	Hydrogen site location: inferred from neighbouring sites
*R*[*F*^2^ > 2σ(*F*^2^)] = 0.039	H-atom parameters constrained
*wR*(*F*^2^) = 0.095	*w* = 1/[σ^2^(*F*_o_^2^) + (0.047*P*)^2^ + ] where *P* = (*F*_o_^2^ + 2*F*_c_^2^)/3
*S* = 1.06	(Δ/σ)_max_ = 0.022
3119 reflections	Δρ_max_ = 0.15 e Å^−^^3^
156 parameters	Δρ_min_ = −0.15 e Å^−^^3^
1 restraint	Absolute structure: Flack (1983), 1074 Friedel pairs
Primary atom site location: structure-invariant direct methods	Flack parameter: −0.03 (8)

### Special details

**Table:**

Geometry. All e.s.d.'s (except the e.s.d. in the dihedral angle between two l.s. planes) are estimated using the full covariance matrix. The cell e.s.d.'s are taken into account individually in the estimation of e.s.d.'s in distances, angles and torsion angles; correlations between e.s.d.'s in cell parameters are only used when they are defined by crystal symmetry. An approximate (isotropic) treatment of cell e.s.d.'s is used for estimating e.s.d.'s involving l.s. planes.
Refinement. Refinement of *F*^2^ against ALL reflections. The weighted *R*-factor *wR* and goodness of fit *S* are based on *F*^2^, conventional *R*-factors *R* are based on *F*, with *F* set to zero for negative *F*^2^. The threshold expression of *F*^2^ > σ(*F*^2^) is used only for calculating *R*-factors(gt) *etc*. and is not relevant to the choice of reflections for refinement. *R*-factors based on *F*^2^ are statistically about twice as large as those based on *F*, and *R*- factors based on ALL data will be even larger.

### Fractional atomic coordinates and isotropic or equivalent isotropic displacement parameters (Å^2^)

**Table:**

	*x*	*y*	*z*	*U*_iso_*/*U*_eq_	
S1	0.48826 (6)	0.23389 (10)	0.70138 (3)	0.05069 (16)	
C1	0.6639 (3)	0.0772 (5)	0.68705 (15)	0.0621 (6)	
H1A	0.7435	0.0945	0.7367	0.075*	
H1B	0.6441	−0.1036	0.6769	0.075*	
C2	0.7188 (2)	0.1937 (4)	0.61568 (12)	0.0502 (5)	
C3	0.6676 (3)	0.1092 (5)	0.53602 (17)	0.0714 (7)	
H3	0.5993	−0.0279	0.5259	0.086*	
C4	0.7162 (3)	0.2252 (7)	0.47099 (14)	0.0778 (7)	
H4	0.6802	0.1663	0.4175	0.093*	
C10	0.3505 (2)	0.0564 (5)	0.91695 (12)	0.0555 (6)	
H10A	0.3303	−0.1252	0.9095	0.067*	
H10B	0.4548	0.0759	0.9492	0.067*	
C9	0.3423 (2)	0.1786 (4)	0.83225 (11)	0.0489 (5)	
H9	0.3620	0.3617	0.8413	0.059*	
C14	0.1834 (2)	0.1520 (4)	0.77427 (13)	0.0571 (6)	
H14A	0.1071	0.2359	0.7996	0.069*	
H14B	0.1862	0.2427	0.7236	0.069*	
C8	0.4742 (2)	0.0711 (5)	0.79491 (12)	0.0516 (5)	
H8A	0.4564	−0.1095	0.7835	0.062*	
H8B	0.5712	0.0884	0.8345	0.062*	
C5	0.8165 (3)	0.4249 (6)	0.48481 (16)	0.0722 (8)	
H5	0.8493	0.5022	0.4409	0.087*	
C11	0.2376 (3)	0.1677 (5)	0.96566 (13)	0.0607 (6)	
H11A	0.2536	0.3509	0.9708	0.073*	
H11B	0.1327	0.1387	0.9354	0.073*	
C7	0.8196 (3)	0.3962 (5)	0.62789 (14)	0.0640 (6)	
H7	0.8557	0.4572	0.6811	0.077*	
C6	0.8688 (3)	0.5117 (6)	0.56292 (15)	0.0737 (7)	
H6	0.9375	0.6484	0.5726	0.088*	
C12	0.2557 (3)	0.0528 (6)	1.05069 (14)	0.0730 (7)	
H12A	0.3602	0.0843	1.0812	0.088*	
H12B	0.2417	−0.1308	1.0455	0.088*	
C15	0.1300 (3)	−0.1175 (6)	0.75268 (15)	0.0782 (7)	
H15A	0.2028	−0.2020	0.7261	0.117*	
H15B	0.0302	−0.1140	0.7161	0.117*	
H15C	0.1228	−0.2083	0.8020	0.117*	
C13	0.1419 (3)	0.1591 (7)	1.09933 (15)	0.0903 (11)	
H13A	0.1537	0.3412	1.1040	0.135*	
H13B	0.1618	0.0843	1.1533	0.135*	
H13C	0.0382	0.1190	1.0713	0.135*	
O1	0.5294 (2)	0.5042 (3)	0.72507 (10)	0.0703 (5)	

### Atomic displacement parameters (Å^2^)

**Table:**

	*U*^11^	*U*^22^	*U*^33^	*U*^12^	*U*^13^	*U*^23^
S1	0.0564 (3)	0.0427 (3)	0.0549 (3)	0.0024 (3)	0.0162 (2)	−0.0007 (3)
C1	0.0675 (15)	0.0432 (14)	0.0836 (16)	0.0064 (12)	0.0342 (13)	0.0086 (12)
C2	0.0520 (11)	0.0406 (15)	0.0624 (12)	0.0054 (10)	0.0215 (9)	0.0006 (10)
C3	0.0696 (15)	0.0641 (17)	0.0844 (17)	−0.0124 (13)	0.0249 (13)	−0.0186 (13)
C4	0.0815 (16)	0.094 (2)	0.0604 (13)	0.006 (2)	0.0211 (12)	−0.0157 (18)
C10	0.0608 (13)	0.0537 (14)	0.0536 (12)	0.0046 (11)	0.0156 (10)	0.0015 (10)
C9	0.0560 (11)	0.0377 (14)	0.0538 (11)	0.0022 (9)	0.0132 (9)	−0.0009 (9)
C14	0.0575 (12)	0.0593 (16)	0.0560 (12)	0.0035 (11)	0.0150 (10)	0.0000 (10)
C8	0.0587 (12)	0.0409 (13)	0.0580 (12)	0.0034 (10)	0.0184 (10)	0.0038 (9)
C5	0.0774 (17)	0.078 (2)	0.0697 (15)	0.0113 (15)	0.0353 (13)	0.0131 (14)
C11	0.0643 (13)	0.0643 (19)	0.0554 (11)	0.0034 (12)	0.0170 (10)	−0.0006 (11)
C7	0.0638 (14)	0.0697 (18)	0.0593 (13)	−0.0104 (14)	0.0145 (11)	−0.0028 (12)
C6	0.0769 (17)	0.0720 (19)	0.0771 (16)	−0.0169 (14)	0.0270 (13)	0.0031 (14)
C12	0.0772 (16)	0.084 (2)	0.0600 (14)	0.0012 (15)	0.0203 (12)	−0.0009 (13)
C15	0.0746 (16)	0.0795 (19)	0.0805 (16)	−0.0213 (16)	0.0164 (13)	−0.0149 (15)
C13	0.0885 (18)	0.122 (3)	0.0676 (15)	0.0008 (18)	0.0328 (14)	−0.0044 (16)
O1	0.1012 (13)	0.0340 (9)	0.0831 (10)	0.0008 (9)	0.0366 (9)	0.0008 (8)

### Geometric parameters (Å, °)

**Table:**

S1—O1	1.4923 (18)	C14—H14B	0.9700
S1—C8	1.797 (2)	C8—H8A	0.9700
S1—C1	1.813 (2)	C8—H8B	0.9700
C1—C2	1.499 (3)	C5—C6	1.360 (4)
C1—H1A	0.9700	C5—H5	0.9300
C1—H1B	0.9700	C11—C12	1.511 (3)
C2—C7	1.372 (3)	C11—H11A	0.9700
C2—C3	1.378 (3)	C11—H11B	0.9700
C3—C4	1.382 (4)	C7—C6	1.382 (3)
C3—H3	0.9300	C7—H7	0.9300
C4—C5	1.358 (4)	C6—H6	0.9300
C4—H4	0.9300	C12—C13	1.520 (3)
C10—C11	1.523 (3)	C12—H12A	0.9700
C10—C9	1.532 (3)	C12—H12B	0.9700
C10—H10A	0.9700	C15—H15A	0.9600
C10—H10B	0.9700	C15—H15B	0.9600
C9—C14	1.534 (3)	C15—H15C	0.9600
C9—C8	1.535 (3)	C13—H13A	0.9600
C9—H9	0.9800	C13—H13B	0.9600
C14—C15	1.507 (3)	C13—H13C	0.9600
C14—H14A	0.9700		
			
O1—S1—C8	106.19 (10)	S1—C8—H8A	109.3
O1—S1—C1	107.14 (11)	C9—C8—H8B	109.3
C8—S1—C1	96.52 (10)	S1—C8—H8B	109.3
C2—C1—S1	110.21 (15)	H8A—C8—H8B	107.9
C2—C1—H1A	109.6	C4—C5—C6	119.9 (2)
S1—C1—H1A	109.6	C4—C5—H5	120.1
C2—C1—H1B	109.6	C6—C5—H5	120.1
S1—C1—H1B	109.6	C12—C11—C10	112.9 (2)
H1A—C1—H1B	108.1	C12—C11—H11A	109.0
C7—C2—C3	117.6 (2)	C10—C11—H11A	109.0
C7—C2—C1	120.3 (2)	C12—C11—H11B	109.0
C3—C2—C1	122.1 (2)	C10—C11—H11B	109.0
C2—C3—C4	121.0 (2)	H11A—C11—H11B	107.8
C2—C3—H3	119.5	C2—C7—C6	121.5 (2)
C4—C3—H3	119.5	C2—C7—H7	119.3
C5—C4—C3	120.3 (2)	C6—C7—H7	119.3
C5—C4—H4	119.9	C5—C6—C7	119.8 (3)
C3—C4—H4	119.9	C5—C6—H6	120.1
C11—C10—C9	114.60 (18)	C7—C6—H6	120.1
C11—C10—H10A	108.6	C11—C12—C13	113.4 (2)
C9—C10—H10A	108.6	C11—C12—H12A	108.9
C11—C10—H10B	108.6	C13—C12—H12A	108.9
C9—C10—H10B	108.6	C11—C12—H12B	108.9
H10A—C10—H10B	107.6	C13—C12—H12B	108.9
C10—C9—C14	113.55 (17)	H12A—C12—H12B	107.7
C10—C9—C8	108.82 (16)	C14—C15—H15A	109.5
C14—C9—C8	112.70 (16)	C14—C15—H15B	109.5
C10—C9—H9	107.1	H15A—C15—H15B	109.5
C14—C9—H9	107.1	C14—C15—H15C	109.5
C8—C9—H9	107.1	H15A—C15—H15C	109.5
C15—C14—C9	115.7 (2)	H15B—C15—H15C	109.5
C15—C14—H14A	108.3	C12—C13—H13A	109.5
C9—C14—H14A	108.3	C12—C13—H13B	109.5
C15—C14—H14B	108.3	H13A—C13—H13B	109.5
C9—C14—H14B	108.3	C12—C13—H13C	109.5
H14A—C14—H14B	107.4	H13A—C13—H13C	109.5
C9—C8—S1	111.68 (15)	H13B—C13—H13C	109.5
C9—C8—H8A	109.3		
			
O1—S1—C1—C2	−64.88 (19)	C10—C9—C8—S1	−172.63 (15)
C8—S1—C1—C2	−174.08 (17)	C14—C9—C8—S1	60.5 (2)
S1—C1—C2—C7	90.3 (2)	O1—S1—C8—C9	63.90 (17)
S1—C1—C2—C3	−87.7 (2)	C1—S1—C8—C9	173.90 (16)
C7—C2—C3—C4	−0.1 (4)	C3—C4—C5—C6	−0.2 (4)
C1—C2—C3—C4	177.9 (2)	C9—C10—C11—C12	−176.65 (19)
C2—C3—C4—C5	0.3 (4)	C3—C2—C7—C6	−0.1 (4)
C11—C10—C9—C14	−60.3 (3)	C1—C2—C7—C6	−178.2 (2)
C11—C10—C9—C8	173.35 (18)	C4—C5—C6—C7	0.0 (4)
C10—C9—C14—C15	−61.5 (2)	C2—C7—C6—C5	0.2 (4)
C8—C9—C14—C15	62.8 (2)	C10—C11—C12—C13	−179.0 (2)

### Hydrogen-bond geometry (Å, °)

**Table:**

*D*—H···*A*	*D*—H	H···*A*	*D*···*A*	*D*—H···*A*
C8—H8A···O1^i^	0.97	2.39	3.258 (3)	149
C1—H1B···O1^i^	0.97	2.49	3.333 (3)	145

Symmetry codes: (i) *x*, *y*−1, *z*.
